# Polinton-like viruses are abundant in aquatic ecosystems

**DOI:** 10.1186/s40168-020-00956-0

**Published:** 2021-01-12

**Authors:** Christopher M. Bellas, Ruben Sommaruga

**Affiliations:** Department of Ecology, University of Innsbruck, Technikerstrasse 25, A-6020 Innsbruck, Austria

**Keywords:** Polinton, Virophage, NCLDV, Metagenome, Lakes

## Abstract

**Background:**

Polintons are large mobile genetic elements found in the genomes of eukaryotic organisms that are considered the ancient ancestors of most eukaryotic dsDNA viruses. Originally considered as transposons, they have been found to encode virus capsid genes, suggesting they may actually be integrated viruses; however, an extracellular form has yet to be detected. Recently, circa 25 Polinton-like viruses have been discovered in environmental metagenomes and algal genomes, which shared distantly related genes to both Polintons and virophages (*Lavidaviridae*). These entities could be the first members of a major class of ancient eukaryotic viruses; however, owing to the lack of available genomes for analysis, information on their global diversity, evolutionary relationships, eukaryotic hosts, and status as free virus particles is limited.

**Results:**

Here, we analysed the metaviromes of an alpine lake to show that Polinton-like virus genome sequences are abundant in the water column. We identify major capsid protein genes belonging to 82 new Polinton-like viruses and use these to interrogate publicly available metagenomic datasets, identifying 543 genomes and a further 16 integrated into eukaryotic genomes. Using an analysis of shared gene content and major capsid protein phylogeny, we define large groups of Polinton-like viruses and link them to diverse eukaryotic hosts, including a new group of viruses, which possess all the core genes of virophages and infect oomycetes and Chrysophyceae.

**Conclusions:**

Our study increased the number of known Polinton-like viruses by 25-fold, identifying five major new groups of eukaryotic viruses, which until now have been hidden in metagenomic datasets. The large enrichment (> 100-fold) of Polinton-like virus sequences in the virus-sized fraction of this alpine lake and the fact that their viral major capsid proteins are found in eukaryotic host transcriptomes support the hypothesis that Polintons in unicellular eukaryotes are viruses. In summary, our data reveals a diverse assemblage of globally distributed viruses, associated with a wide range of unicellular eukaryotic hosts. We anticipate that the methods we have developed for Polinton-like virus detection and the database of over 20,000 genes we present will allow for continued discovery and analysis of these new viral groups.

**Video abstract**

**Supplementary Information:**

The online version contains supplementary material available at 10.1186/s40168-020-00956-0.

## Introduction

Polintons (also known as Mavericks) are the largest transposable elements known in eukaryotic genomes. Most are between 15 and 40 kilobase pairs (kb) in length [1], encoding a protein-primed type B DNA *POL*ymerase (pPolB) and a retroviral-like (RVE family) *INT*egrase gene which gives rise to their name [2]. Recently, many Polintons in animal genomes have been found to additionally encode virus hallmark genes, including a major capsid protein (MCP), minor capsid protein (mCP), and a DNA packaging ATPase [3], suggesting they are actually integrated viruses, in which case they would be reclassified as Polintoviruses [3]. To date, no extracellular Polintons have been observed; however, they are similar in size and genomic architecture to the virophages, a group of viruses which infect microbial eukaryotes, but are entirely dependent on co-infecting nucleocytoplasmic large DNA viruses (NCLDV) or ‘giant viruses’ to carry out their replication [4]. The virophage Mavirus in particular appears to be related to Polintons [5], possessing the core Polinton pPolB and RVE integrase genes and an ability to integrate into the genome of its eukaryotic host, *Cafeteria roenbergensis*, where it acts as an antivirus defence system against infection by the giant virus CroV [6]. Mavirus is a bona fide virus, but in its integrated form in the *C. roenbergensis* genome, it resembles a Polinton; however, its MCP gene is clearly related to the virophages, suggesting that virophages evolved from Polintons [7]. The recent discovery of ca. 25 Polinton-like viruses (PLVs) in sequencing datasets and environmental metagenomes [8–10] has further revealed a broad assemblage of related viruses, several of which were found integrated into algal genomes or associated with (attached to or inside) NCLDV particles [9, 10]. PLVs are similar in size and genomic composition to the Polintons, but to date they lack the capsid maturation protease gene of Polintons and virophages and rarely contain pPolB or RVE genes. Known PLVs are only distantly related to Polintons at the protein sequence level, and their double jelly-roll fold MCP genes are highly distinct from Polintons, which means they are considered a separate group of viruses. Only one PLV, *Tetraselmis striata* virus (TsV-N1), has been isolated as a virus particle. TsV-N1 has a 31-kb genome and is estimated to release thousands of 60 nm virions per algal host cell [11]. This points to the existence of PLVs as free viruses in aquatic ecosystems.

In this study, we report the discovery of abundant eukaryotic viruses in an alpine lake with small (< 41 kb) dsDNA genomes; their predicted genes produce few hits with known proteins by searching against the GenBank non-redundant protein database, but by annotating using remote protein homology detection tools, we find they are related to Polintons, Polinton-like viruses, and virophages. Using their novel MCP genes as bait to interrogate publicly available metagenomes, we build a database of 666 PLV genomes and perform an analysis of shared gene content to show that this assemblage forms at least eight distinct groups of viruses, which can be linked to diverse unicellular eukaryotic linages.

## Results

### Discovery of novel Polinton-like viruses

**Fig. 1:**
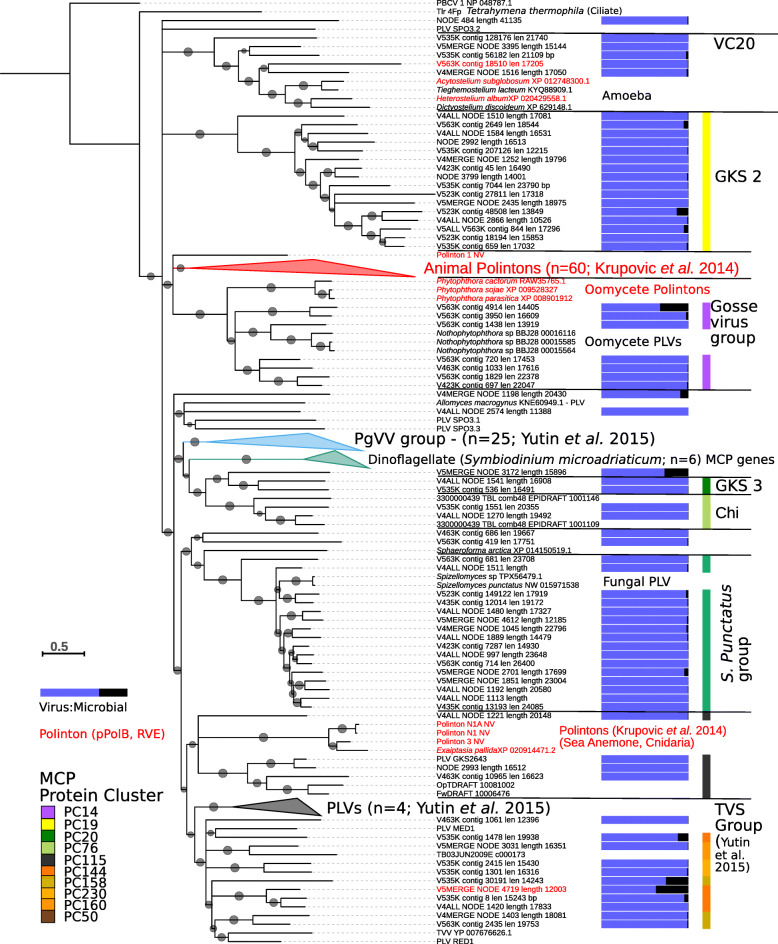
Phylogenetic analysis of the major capsid protein gene of Polinton-like viruses and Polintons. A maximum likelihood tree constructed using MCP genes from Gossenköllesee PLVs, known Polintons and related MCP genes in eukaryotic genomes. Grey circles represent SH (Shimodaira–Hasegawa)-like local support values of between 50 and 100%. Branches with < 50% support were collapsed. Viruses with a confirmed Polinton genomic configuration (pPolB and RVE integrase) are labelled in red. The bar represents the ratio of normalised reads recruited from the viral (blue) and microbial (black) fraction metagenomes (Gossenköllesee only). The colour key represents the assigned protein cluster for the MCPs based on the network-based analysis. Annotated groups show the network analysis-based clusters from Fig. 3, or previously defined MCP groups

To investigate virus diversity in an oligotrophic, alpine lake [12], we created paired virus-size fraction (< 0.2 μm) and microbial metagenomes (> 0.2 μm) from the high mountain lake, Gossenköllesee (2417 m above sea level, Austria) generating 127 GB of metagenomic sequencing data from three time points, October 2017 and February and April 2018, which was assembled separately for each time point. We identified 32 novel virophage genomes in Gossenköllesee, which possessed the characteristic virophage type MCP gene that defines this virus group [13] (Additional file 1: Table S1). Additionally, we identified many relatively abundant, similar sized (10–41 kb) virus contigs that shared several homologous genes with virophages, but possessed no detectable virophage MCP gene. Annotation of these contigs via HHpred against the Protein Data Bank [14] allowed us to identify an ATPase, mCP, and an MCP possessing a double jelly-roll fold that had a distant homology to MCP genes of giant viruses (Additional file 1: Table S2). A similar double jelly-roll fold MCP gene has previously been identified in Polintons and Polinton-like viruses (PLVs) [3, 8] leading us to conclude these were related entities. In total, we discovered 82 new PLVs in Gossenköllesee (see the ‘Materials and methods’ section), many of which appeared to represent complete genomes based on the presence of terminal inverted repeats (TIRs) or a circular mapping genome (Additional file 1: Table S3). Polintons, virophages, and PLV often end in TIRs [2, 8]. Hence, their detection or the detection of a circular genome suggests a complete entity. We also detected 16 related elements in eukaryotic genomes, one in the soil fungus *Spizellomyces punctatus* and 15 complete genomes that were retrieved from oomycetes (Additional file 1: Table S4). In the fungal genome and ten oomycetes, these elements were integrated into the middle of large contigs of the eukaryotic genome, and in seven of these, we detected terminal inverted repeats at the end of the insertion sequence, three of which were flanked by target site duplications of 5–7 bp. These repeats were in entities possessing tyrosine recombinase or RVE integrases (Additional file 1: Table S4). To determine the relationship between the PLVs found in Gossenköllesee and known entities, we constructed a maximum likelihood tree of MCP genes, including those identified in known Polintons (*n* = 56) and PLV (*n* = 25) [8] along with more distantly related homologues from GenBank, which we identified via an iterative PSI-BLAST search based on our new capsid genes (see the ‘Materials and methods’ section). Maximum likelihood phylogenetic analysis of PLV MCP genes showed that most of the Gossenköllesee MCPs formed novel groups, several of which had little homology to previously described Polinton or PLV capsid genes (Fig. 1). These included a group that we refer to as the Gossevirus group, composed of seven Gossenköllesee PLVs and homologous MCPs from Polintons/PLVs detected in oomycete genomes; the GKS2 group, which was composed of 16 Gossenköllesee MCP genes with no known integrated representatives; and the VC20 group, a novel group of five viruses from Gossenköllesee, which possessed MCP genes also detected in four different species of amoeba. In two of the entities in amoeba, we detected pPolB and RVE genes within 10 kb of the MCP gene, suggesting they had a Polinton genomic configuration. An alignment of the MCP genes from the VC20 group used as a HHpred query detected distant homology to Singapore grouper iridovirus MCP (90.2% confidence). Most previously known animal Polinton MCP genes formed one large group, which also contained five MCP genes from Gossenköllese. Six unique virus MCP genes were found in the dinoflagellate *Symbiodinium microadriaticum*; however, we were unable to annotate further PLV genes indicating they may be degraded elements, and we also identified novel MCP genes in the ichthyosporean *Sphaeroforma arctica* and in the fungus *Allomyces macrogynus*. In the latter case, we confirmed the presence of several PLV genes (MCP, ATPase, DNA helicase) implying these were integrated viruses.

### Polinton-like viruses are abundant in the virus-size fraction

**Fig. 2:**
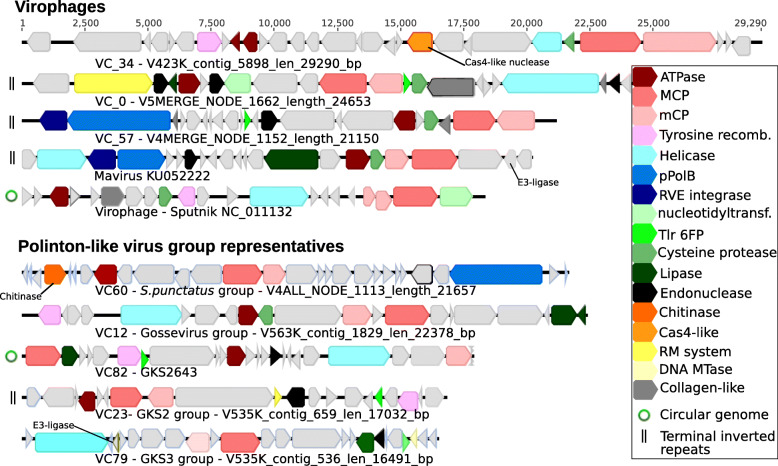
Genome annotations of PLVs and virophages. PLV and new virophage representatives from seven of the major virus groups are displayed. The VC_34 virophage represents a new virus cluster (*n* = 12) of which all members possess a Cas4-like nuclease. PLV_GKS2643 was ungrouped, but represented the most abundant PLV in Gossenkӧllesee

PLVs were present in all three sampling times in Gossenköllesee (October 2017 and February and April 2018); however, their highest relative abundance occurred in the ice-free sampling period (October), when up to 4.9% of all metagenomic reads from the virome could be mapped back to PLVs (Additional file 1: Table S3). Several of the PLVs in Gossenköllesee were highly represented in the viromes and were comparable to the most abundant bacteriophages, including one 17.8-kb circular contig (Fig. 2) (PLV_GKS2643), which was the 8th most abundant virus contig in the entire virome (1193 × coverage per Gb; relative abundance based on normalised depth of recruited reads). Relative metagenomic recruitments to the 83 PLVs vs 23 virophages (contigs > 10 kb) were 32 Mb Gb^−1^ vs 2 Mb Gb^−1^ of metagenomic data, each of the top three most abundant PLVs individually recruited more reads than the combined virophage population.

To address whether Gossenköllesee PLVs are genuine-free virus particles or the detection of integrated elements in eukaryotic genomes, we compared the number of normalised metagenomic reads that mapped to our PLVs from the viral and microbial size fractions. We found that most (84%) of the PLVs in our database recruited 95% or more reads from the viral size fraction (Additional file 1: Table S3) (median 190-fold enrichment in the viral size fraction; range 1–2816-fold). This was similar to virophages (median 108-fold enrichment; range 1–1078) supporting the idea that PLVs are bona fide viruses. Five contigs with low representation in the virus-size fraction (0–5 × coverage Gb^−1^) possessed MCP genes homologous to those found in animal Polintons. Animal Polintons have a distinct MCP gene (Fig. 1) and are currently only known as integrated elements [3]. Our analysis revealed that these five contigs were the only ones enriched in the microbial fraction (50–450-fold), consistent with this group being mainly integrated elements and supporting our approach (Additional file 1: Table S3). Three further PLVs and one virophage also had a relatively strong signal in both the viral and microbial size fractions (Fig. 1, Additional file 1: Table S3), which would be expected if they are capable of existing as free virus particles and integrated elements. Our viromes were generated from 0.2 μm filtered, DNase-treated samples (see the ‘Materials and methods’ section), which should remove most eukaryotes and bacteria whilst digest contaminating (non-encapsidated) free DNA; however, non-viral reads may remain in viromes despite extensive purification [15]. Only low-level eukaryotic contamination could be detected in our dataset by searching against the SILVA SSU (16S/18S rRNA) database (BLASTP *E* value < 10^−5^) in 4 contigs (0.2–1 × coverage Gb^−1^), with the most abundant hit being to a chloroplast (Additional file 1: Table S5). The large relative abundance of PLVs in our dataset precludes the possibility that eukaryotic DNA contamination in our viromes could account for their detection if they are low copy number transposons (sequencing a eukaryotic genome at 100s to 1000s of times coverage would require many times the sequencing effort than we applied to our entire viromes). The most abundant circular PLV genome was sequenced at 1193 × coverage Gb^−1^, in a 70-Gb virus metagenome (~ 80,000-fold coverage), 1100-fold higher relative recruitment than in the microbial size fraction. Considering that 82 new PLVs were assembled into contigs which were always less than 41 kb (the maximum sized circular PLV we discovered), PLVs were always enriched in the virus-size fraction and together they recruited up to 4.9% of all metaviromic reads; this supports the idea that they are virus particles.

### Expanding global Polinton-like virus diversity

To determine whether the novel PLVs we found were widespread in other aquatic ecosystems, we used all MCP genes as bait to interrogate publicly available metagenomes and build a database of global PLV diversity. A profile Hidden Markov Model (profile HMM) was built from the alignment of all new and known PLV MCP genes using HMMER (hmmer.org). This was used to interrogate the Integrated Microbial Genomes Virus (IMG/VR) database [16] of globally derived viral sequences (retrieving 167 PLVs) from terrestrial, marine, and predominantly freshwater ecosystems. The IMG/VR database was originally built from NCBI RefSeq and automatically identified virus contigs from IMG microbial metagenomes and metaviromes. Hence, IMG/VR sequences come from all size fractions and we cannot exclude the possibility that some PLV from this dataset could come from integrated elements. However, the greatest number of IMG/VR hits came from two viromes, Lake Soyang and Han River (South Korea) [17, 18]. These viromes were highly purified by the authors using CsCl gradient ultracentrifugation, and only buoyant densities corresponding to dsDNA viruses were collected. This means it is highly likely that the entities detected from these viromes were virus particles. To enhance PLV detection in these two metaviromes, they were downloaded, reassembled (see the ‘Materials and methods’ section), and interrogated (BLASTP; *E* value cutoff 10^−5^) retrieving 376 further PLVs. Hence, we detected and retrieved 625 new PLV genomes over 10 kb from predominantly freshwater metagenomes (Additional file 1: Table S4), which represents a 25-fold increase in known genomes. To build a dataset of related virus genomes for comparison, we added 25 known PLVs (Table 1) at the time of analysis (February 2019) along with the 15 integrated elements from oomycetes and one from *S. punctatus*. We also added 374 virophage genomes to our dataset, 239 of these were previously described or detected genomes, including 200 from a large, detailed analysis of global virophage diversity in metagenomes [13], whilst 135 were novel genomes (Table 1). As virophages have been the subject of extensive analysis, their inclusion in our analysis was to determine their shared gene content with the PLV groups.

**Table 1:**
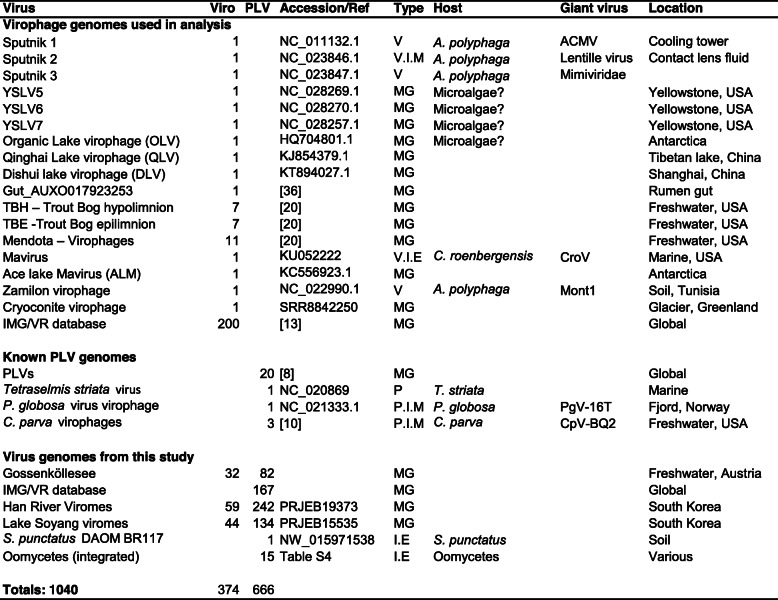
PLV and virophage contigs used and detected in this study

Viro, virophage; PLV, Polinton-like virus. Type: V, virophage; P, Polinton-like virus; I, integrated; E, eukaryote integration; M, Megavirales integration; MG, metagenomic detection only. *C. parva*, *Chrysochromulina parva*; *P. globosa*, *Phaeocyctis globosa*; *C. roenbergensis*, *Cafeteria roenbergensis*; *A.polyphaga*, *Acanthamoeba polyphaga*; CroV, *Cafeteria roenbergensis* virus; *S. punctatus*, *Spizellomyces punctatus*

**Fig. 3:**
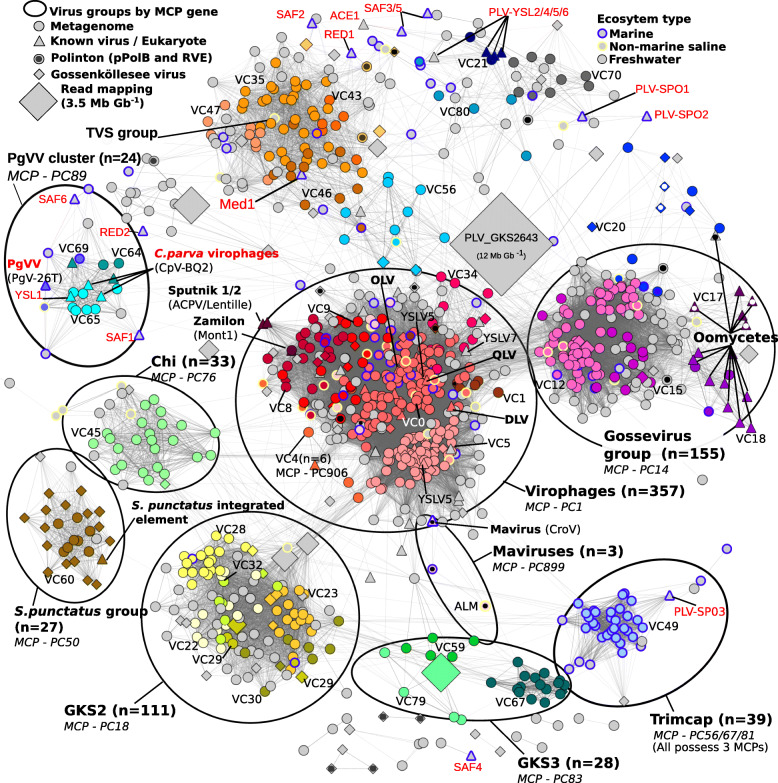
Polinton-like viruses (PLVs), Polintons, and virophages share a network of genes. A network-based analysis of shared protein clusters from 1015 virophage, PLV, and Polinton genomes visualised in Cytoscape. Each symbol (node) represents a complete or near complete virus genomes of 10–41 kb; the lines (edges) represent the strength of the connectivity (edge weight) between each genome. Node colours represent virus clusters (VCs) defined by vConTACT2 with 5 or more members or with a known isolate, and each VC is labelled in black next to the corresponding colour group. Symbols represent virus origin: Gossenköllesee (GKS) metagenomes (diamond), isolated/previously detected viruses (triangle), and retrieved from global metagenomes (circle). Polintons containing pPolB and RVE integrase include a black or white circle inside the symbol. The size of the diamond symbols represents the metagenomic read recruitment from Gossenköllesee (Mb per Gb metagenome—see scale top left). Known isolated viruses and integrated elements are labelled in bold with associated giant virus (if known) in brackets. Circles show larger virus groups sharing the same major capsid protein (MCP) cluster. Previously known PLV genomes are highlighted with a red label. Viruses without shared protein clusters (*n* = 25) are not present in the network. Virus name abbreviations can be found in Table 1

We used a network-based analysis of shared protein clusters from 1040 viruses using vConTACT v.2.0 [19]. Such an all-vs-all gene comparison is suitable when faced with numerous distantly related viruses, which may undergo frequent genetic exchange, allowing new relationships and shared gene content to be detected that complements the MCP phylogeny. Network analysis grouped 739 viruses into 61 virus clusters (VCs) with ≥ 3 members (in bacteriophages, this is equivalent to genus level groupings; however, this remains untested for eukaryotic viruses) and 64 viruses into VCs with only 2 members per cluster (Fig. 3). A further 213 viruses were outliers and 24 were singletons (sharing no protein clusters and not included in the network), including *Tetraselmis striata* virus (TsV-N1) [11], which infects a microalgae of the Chlamydomonadaceae family. The lack of clustering of singletons and outliers suggests a large-scale under-sampling of the true PLV diversity. From the network, it was apparent that individual VCs and outliers formed larger scale virus groupings, which were consistent with the MCP phylogeny (Fig. 1) and the assigned protein cluster (PC) for each MCP (Additional file 1: Table S4). A similar finding was noted in a large study of virophage genomes, where phylogeny of core genes agreed with virus groups based on shared protein clusters [13]. From our analysis of all PLV genomes, PLVs formed eight major groups, five of which were based on Gossenköllesee MCP genes (Gossevirus group, GKS2, GKS3, *S. punctatus* group, and the Chi group). At the level of protein-protein comparisons used in our analysis (BLASTP *E* value < 10^−4^), all virophages formed a single large group, possessing the same virophage type MCP gene (PC_0001) and composed of 11 distinct VCs (Fig. 3), except the Mavirus-like viruses which fell into a related cluster (VC3, *n* = 3), possessing an MCP and other genes which were distinct from the other virophages [20]. The clustering of all virophages into one group serves to highlight the large genomic diversity among the PLVs; several PLV groups shared more detectable protein clusters with virophages than they did with each other. For example, there was considerable genetic overlap between the new Gossevirus group of PLV (*n* = 155) and the virophage cluster. Both groups shared lipase (PC_0008), phospholipase (PC_0010), endonuclease (PC_0013), and tyrosine recombinase (PC_0000) protein clusters (Additional file 1: Table S6); additionally, 90 Gossevirus group members also possessed a cysteine protease gene (PC_0019, PC_0220, PC0492) (Fig. 2), known to be involved in virophage capsid maturation and notably absent from the PLV until now [8]. Gossevirus group members are a distinct group of viruses, possessing a novel MCP gene (PC_0014), which has no detectable BLASTP homology to the virophages. However, multiple sequence alignment with related genes and annotation by HHpred confirmed it as a double jelly-roll fold capsid gene (Additional file 1: Table S2). These MCP genes were also present in integrated elements in oomycete genomes (Fig. 1), which clustered into the Gossevirus group using gene sharing networks (Fig. 3) and suggested a related eukaryotic host for members of this virus group.

The *S. punctatus* integrated PLV clustered into the titular group (*n* = 27) (Fig. 3). This early diverging chytrid fungus is noted for the possession of motile zoospores [21]. Concurrently, in 52% of the viruses in this group, we detected a chitinase gene (Additional file 1: Table S6; Fig. 2), which may be an adaptation for the dissolution of fungal cell walls, suggesting that members of this cluster are able to infect and integrate into fungal genomes, and hence, they may be dsDNA fungal viruses. Additionally, 78% of the viruses from the Chi group (*n* = 32) also contained a chitinase gene (Additional file 1: Table S6), though we did not detect integrated representatives. Network analysis also expanded two known groups of PLV, the PgVV and TVS groups [8]. The PgVV group includes *Phaeocystis globosa* virus virophage (PgVV) and three *Chrysochromulina parva* virus virophages, which despite their names are all PLVs [8, 10] (Table 1); all four known members share the same MCP protein cluster (PC_0089) and are associated with giant viruses infecting haptophytes (Table 1). We expanded this group with a further 20 representatives from global metagenomes; however, no new PgVV group members were detected in Gossenköllesee. The TVS group formed a large, loosely defined virus cluster in our network analysis, which was composed of four related MCP protein clusters, 11 Gossenköllesee PLV clustered within this group (Fig. 1). A final well-defined group of viruses that we called the Trimcap group (Tri-major capsid) formed around one Gossenköllesee member and a unique PLV from a previous study (PLV-SP03) [8]. All 39 members of the Trimcap group possessed the unusual configuration of encoding three distinct MCP genes.

### Genomic content of the Polinton-like viruses

All PLVs contained the three core genes MCP, mCP, and DNA packaging ATPase; however, each PLV group contained a highly diverged version of the MCP and ATPase genes, with mCP genes being highly variable even within a PLV group. Each PLV group also had a highly variable gene content between virus clusters, for example, Gossevirus group members in VC_12 contained cysteine protease as a core gene (65 of 67 members) (Additional file 1: Table S7), whereas VC_15 Gossevirus group members shared the same MCP and ATPase protein clusters but possessed a hypothetical protein instead of the protease (13 of 13 members). Most PLV genes remained as hypothetical proteins, even after HHpred analysis; however, many PLVs were found to encode genes, which may be involved in countering host defence mechanisms against viral infection. As has been documented for virophages [13], over half of all viruses in our dataset, both PLV and virophages (*n* = 632) encoded a DNA methyltransferase gene. Additionally, with sensitive homology detection, we detected at least 7 virophages from VC_0 and VC_4 which possessed putative restriction modification systems (type 1 or type III restriction enzyme linked to a DNA methyltransferase) (Additional file 1: Table S7), previously hypothesised to aid giant viruses in their defence against virophages [4]. Similarly, 61 viruses in our dataset across all virus groups (apart from the *S. Punctatus* and Chi groups) contained a predicted E3 ubiquitin-protein ligase. E3 ligases are known to counter host immune defences in vertebrate viruses [22] and have previously been detected in one other PLV [10].

### Predicting eukaryotic hosts

**Fig. 4:**
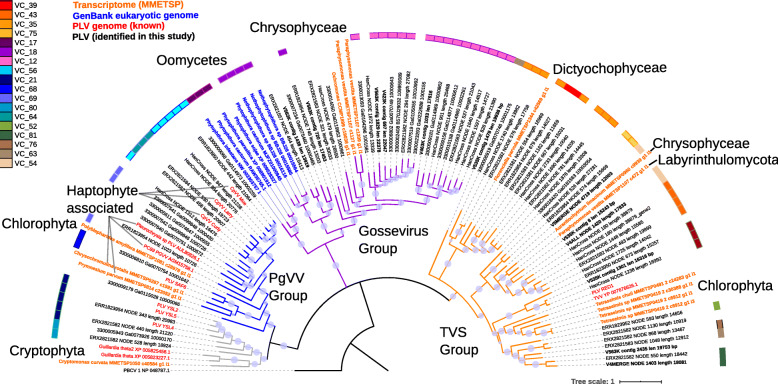
Eukaryotic hosts transcribing PLV major capsid genes and their relationship to the virus groups. Maximum likelihood phylogenetic analysis of PLV major capsid protein genes found in eukaryotic transcriptomes (orange labels), eukaryotic genomes (blue), along with MCP genes from metagenomically detected (black) or previously known (red) PLV, generated as per Fig. 1. Transcriptome MCP genes were related to PLV found in the PgVV, TVS, and Gossevirus groups only (Fig. 3). *Paramecium bursaria* Chlorella virus 1 (PBCV 1) was used as an outgroup. Virus clusters refer to those defined by the network analysis in Fig. 3

The initial discovery of PLVs identified several algal hosts for these viruses based on their genomic integration or the detection of related MCP genes, and these were in the cryptophyte *Guillardia theta*, the chlorophytes *Tetraselmis striata* and *Monoraphidium neglectum* [8], and later in five PLVs, which were found associated with giant viruses infecting haptophytes [9, 10]. Our analysis has demonstrated that viruses related to the novel groups found in Gossenköllesee can also be found in amoeba (VC20 group), oomycetes (Gossevirus group), and fungi (*S. punctatus* group). The phytoplankton of Gossenköllesee and other alpine lakes is usually dominated by dinoflagellates and chrysophytes [23], which also makes these groups strong host candidates for some of the most abundant, unassigned, PLV in the lake. To improve detection of eukaryotic host groups, we searched for PLV MCP genes in 680 transcriptomes (see the ‘Materials and methods’ section) from the Marine Microbial Eukaryotic Transcriptome Sequencing Project (MMETSP) [24], detecting 14 MCP genes in 10 different eukaryotic transcriptomes. The detection of MCP genes in transcriptomes has previously been used to successfully detect virophage infections in marine algae [13]. Phylogenetic analysis of the transcriptome MCP genes placed three genes in the new Gossevirus group (Fig. 4), and these MCP genes came from Chrysophyceae (*Ochromonas* sp. and two MCPs in *Paraphysomonas vestita*) suggesting that some members of the Gossevirus group of PLV are capable of infecting the abundant Chrysophyceae in the lake. Both oomycetes and Chrysophyceae belong to the Stramenopile group of eukaryotic organisms, which suggests the Gossevirus group of PLV are associated with this group; however, the *P. vestita* transcriptome contained a third, highly divergent MCP gene which was placed in the TVS group of PLV, showing that PLV from highly different groups can infect the same hosts. Six other transcriptome MCP genes were placed in the TVS group; four were detected in *Tetraselmis* sp., one in *Florenciella parvula*, and one in *Aurantiochytrium limacinum*, confirming the TVS group viruses are hosted by a diverse range of eukaryotic lineages. PLVs found in Gossenköllesee were most closely related to *P. vestita* and *A. limacinum* in the TVS group (Fig. 4). Three other transcriptome MCP genes belonged to the PgVV group of PLV, *Chrysochromulina rotalis*, *Prymnesium parvum*, and *Polyblepharides amylifera*, adding further haptophyte and chlorophyte hosts to this group of viruses. In summary, this analysis of eukaryotic hosts demonstrated that PLVs are associated with a wide range of eukaryotic linages, and some groups of PLV appear to be associated with specific groups, for example, 7 of 8 linkages in the PgVV group are to haptophytes and all 11 host linkages to the Gossevirus group are to Stramenopiles; however, there are several exceptions. Multiple PLV can infect the same host (as it was the case for oomycetes and *P. vestita*), which suggests that opportunities for genetic exchange are frequently encountered among the PLV groups.

## Discussion

This study establishes a major new assemblage of eukaryotic viruses as an important component of many freshwater viromes. The discovery of new groups of globally distributed PLVs raises many questions as to their role in aquatic ecosystems and their place in eukaryotic virus evolution. PLVs may be independently replicating viruses which cause lysis in a eukaryotic host, as reported for *Tetraselmis striata* virus [11]. Alternatively, they may depend on giant viruses for their replication in a manner similar to the virophages. Four members of the PgVV group of PLV were originally found either attached to or inside giant virus virions [9, 10], supporting the idea that they too are dependent on giant viruses for their replication. The genetic overlap between several PLV groups and virophages (Fig. 3) raises the possibility that many more of the PLVs identified here could be giant virus-associated entities, potentially maintained in eukaryotic genomes to attenuate future giant virus infections [2]. Whilst we detected nucleocytoplasmic large DNA viruses (NCLDV) MCP genes in our viromes, we did not detect any of these contigs that shared genes with the PLVs, as was the case for organic lake virophage and its giant virus host [25]. Hence, it is essential that future studies address the relationships of PLVs and NCLDVs in aquatic ecosystems.

PLVs are currently considered to be a separate group of viruses to Polintons known in eukaryotic genomes, owing to their highly diverged MCP genes and their lack of pPolB and RVE genes. The identification of degenerated pPolB motifs in PLV helicases has led to the proposal that PLVs evolved from Polintons [26]. However, with a 25-fold increase in PLV genomes provided by our study, the distinction between Polintons and PLVs in unicellular eukaryotes has been blurred, if Polintons are defined by their gene content alone. For example, 28 viruses in our network fulfilled the definition of Polintons (pPolB, RVE, MCP, mCP, and ATPase). These viruses populated at least 8 virus clusters, spread across the virophages, the TVS group, VC20 group, several outlier viruses, and the Gossevirus group (Fig. 3); hence, viruses with highly divergent MCP genes had Polinton gene configurations (Fig. 1). In the case of the integrated elements in oomycete genomes, four possessed the characteristic Polinton RVE and pPolB gene, three more possessed one of the two genes, whilst five further oomycete elements instead encoded a DNA helicase-primase and tyrosine recombinase, which are more typical of the PLV [8]. Two ‘Polintons’ that we detected in amoeba (*Acytostelium subglobosum* and *Heterostelium album*) also shared a related MCP gene to the VC20 group of PLV (Fig. 1), which again was highly diverged from previously known Polinton MCP genes. The first described Polintons were mainly found in animal genomes and contained the characteristic pPolB and RVE genes which defined the Polinton group [27, 28]; these were later found to encode virus MCP genes [3] (Fig. 1). Further Polintons were found in animals based on these original genomes [1]; however, several early described Polintons from unicellular eukaryotes had no detectable MCP gene, such as those from the fungus *Glomus intraradices* (*Rhizophagus irregularis*), the oomycete *Phytophthora infestans*, the trichomonad *Trichomonas vaginalis*, and the amoeba *Entamoeba invadens*, leading to the idea that some had lost the viral MCP gene to lead a transposon lifestyle [2]. Analysis of the viral forms in Gossenköllesee has allowed us to identify the missing viral MCP gene in *P. infestans* as belonging to the Gossevirus group of PLV (PC_0014). Additionally, we identified a new virus MCP gene in amoeba (VC20 group) and a new MCP gene in the fungus *S. Punctatus* (albeit not yet an MCP in *E. invadens* or *G. intraradices*). Therefore, some of the original ‘Polintons’ now fit into PLV groups. If we define virus groups by their MCP gene phylogeny (Fig. 1) and gene sharing networks (Fig. 3), rather than by the presence or absence of pPolB and RVE genes, there is little distinction between Polintons and PLVs in unicellular eukaryotes. This assemblage of viruses, thus, forms at least eight groups of eukaryotic viruses with the original animal Polintons, which generally possess a phylogenetically distinct MCP gene (Fig. 1), now being proposed as a further distant group of viruses [29].

## Conclusions

Our study has increased the known diversity of Polinton-like viruses significantly, with the identification of 641 new genomes. We show that PLVs are now a major group of globally distributed eukaryotic viruses which can be linked to diverse eukaryotic hosts. We show that PLV MCP genes are present in eukaryotic transcriptomes, which provides conclusive host linkages for these PLVs and implies that these organisms are transcribing and building viral capsid genes for the release of PLV as extracellular viruses. These are likely the same viral forms that we detected in our virus metagenomes. Most of the new virus genomes in this study were based on an extensive analysis of a single alpine lake virome, using the newly identified MCP genes to interrogate global metagenomes. This is probably why most PLV genomes we detected in global metagenomes came from freshwater ecosystems. As PLVs have previously been detected in marine environments [8, 9, 11], and new MCP gene variants can only be detected by distant protein homology detection tools, it is therefore likely that many more genomes remain hidden in sequencing datasets. Hence, the PLVs we present here probably represent only a small fraction of their true global diversity and they are not necessarily more abundant in freshwaters. Detailed analysis of different ecosystems with different potential host populations should reveal many further groups of these eukaryotic viruses.

## Materials and methods

### Sampling and metagenome generation

We sampled the water column of an oligotrophic, high mountain lake (2417 m), Gossenköllesee, Austria (47° 13′ 46.7″ N, 11° 00′ 47.9″ E), in October 2017 and February and April 2018 to generate paired virus size (< 0.2 μm) and microbial size fraction (> 0.2 μm) metagenomes. For the virus metagenomes and for each sampling time, 40 L composite lake water samples (*n* = 3) were collected from 1 to 8 m depth and passed in sequence through a 142-mm GF/A (Whatman WHA1820150) then a 142-mm 0.22-μm PES filter (Millipore GPWP14250). Virus concentration and purification were by iron chloride precipitation [30], using 4 mL of 10 g L^−1^ Fe stock solution to treat each 40 L replicate. The precipitate was filtered onto three 1-μm pore size polycarbonate filters of 142 mm diameter (Whatman WHA112110) per replicate and stored at 4 °C in separate 50-mL tubes until further processing. The iron-virus precipitate was digested to release virus particles using 40-mL ascorbate-EDTA buffer [30] (0.2 M MgCl_2_, 0.1 M EDTA, 0.2 M ascorbate buffer, pH 6) per filter. The resulting mixture was further filtered through a 47-mm 0.22-μm PES membrane (Millipore GPWP04700) before being treated with DNase I (100 U/mL) for 2 h (DNase stock solution 1000 U/mL in 10 × reaction buffer: 25 mM MgCl_2_, 5 mM CaCl_2_, 100 mM Tris-HCl pH 7.5) and concentrated to ~ 2 mL using two 100-kDa Amicon Ultra-15 Centrifugal Filter Units (Merck-Millipore UFC910024). DNA was extracted from the mixture using 4 × Promega Wizard® Minicolumns per replicate to extract DNA with a ratio of 0.5:1 virus concentrate to Wizard® PCR Preps DNA Purification Resin (Promega: A7170), and the DNA was eluted from each column using 200 μL of 1 × TE buffer (pH 8). The resultant crude extract (~ 800 μL per replicate) was cleaned and further concentrated to make it suitable for Illumina library preparation by firstly ethanol precipitating the DNA (0.3 M NaCl and 2 μL glycogen—Invitrogen 10814010) and resuspended in 100 μL 1 × TE buffer (pH 8). The concentrate was then washed via diafiltration using Amicon Ultra-2 mL Centrifugal Filter units (30 kDa cutoff). Diafiltration consisted of adding 2 mL of wash buffer and spinning down at 2000×*g* until the sample volume reached ~ 100 μL. The filtrate was discarded and the washing step repeated. The diafiltration steps were 3 × rounds of wash buffer 1 (100 mM EDTA, 300 mM NaCl, 10 mM Tris-Cl pH 8), 2 × rounds of wash buffer 2 (300 mM NaCl and 10 mM Tris-Cl pH 8), and 2 rounds of 1 × TE buffer (pH 8). The resultant washed DNA (100 μL) was then cleaned again with a DNeasy PowerClean Pro Cleanup Kit (Qiagen 12997-50).

To extract DNA from the microbial size fraction, 1 L of composite lake water sample per time point (*n* = 1) was filtered onto a 0.2-μm polycarbonate membrane (47 mm, Whatman GPWP04700) which was frozen until further processing. DNA was extracted from the filter (Acinas lab DNA extraction method) [31]. Briefly, filters were cut up and digested in a lysis buffer (40 mM EDTA, 50 mM Tris-HCl pH 9, 750 mM sucrose, 1 mg/mL lysozyme) for 45 min at 37 °C, then 55 °C for 60 min with the addition of SDS (1% final conc.) and Proteinase K (0.2 mg/mL). DNA was extracted with a standard phenol:chloroform:isoamyl alcohol (25:24:1, v/v) protocol with 3 × chloroform:isoamyl alcohol washes (24:1). Residual phenol was further removed from the sample by cleaning with a DNeasy PowerClean Pro Cleanup Kit (Qiagen).

Sequencing libraries for all samples were prepared by the Bristol Genomics Facility (http://www.bristol.ac.uk/biology/genomics-facility/) using the TrueSeq nano LT library prep kit and sequenced on an Illumina NextSeq 500 using 2 × 150 bp paired end reads on two high output runs.

### Assembly and gene prediction

Raw reads were trimmed for Illumina adapters using Trimmomatic [32] before each replicate metagenome was assembled separately using SPAdes [33] version: 3.12.0 (settings: --meta -k 21,33,53,77). A pooled assembly for each virome was also created for each time point by concatenating the 3 replicate fasta files into one assembly. Genes were predicted on all assembled virus contigs > 5 kb using GeneMarkS [34]. Putative complete genomes, i.e. circular contigs, or those with terminal direct or inverted repeats were identified via read mapping with Bowtie2 [35] (--sensitive --no-unal -I 0 -X 800). The resulting SAM file was searched for contigs where discordantly mapping reads over 10 kb were within 1000 bp of each end of the contig, and their coverage depth was > 10% of the mean contig coverage. We flagged such contigs as a circular genome or a genome possessing terminal inverted repeats. Identification of a circular contig or the possession of terminal inverted repeats is an established procedure for flagging virophage genomes as complete [13, 20].

### Detecting virophages and PLVs in Gossenköllesee

To detect virophages in our dataset, we downloaded all virophage MCP genes available from GenBank (December 2018) and concatenated these with those from two other recent metagenomic studies [20, 36]. This file was used to interrogate the predicted genes from the pooled metagenomic assemblies (BLASTP *E* value cutoff 10^−5^). Amino acid sequences from a subsample of these putative virophages (*n* = 13; Additional file 1: Table S1) were annotated by HHpred [37] for remote protein homology detection (https://toolkit.tuebingen.mpg.de/#/tools/hhpred). Genes from these confirmed virophages were then pooled with all virophage genes to create a custom database which was used to search all our metagenomic assemblies for further distantly related virophage-like contigs (BLASTP *E* value cutoff 10^−5^). A putative PLV was flagged where the following criteria were all met: (1) ≥ 20% of predicted genes on a metagenomic contig hit to the custom virophage gene database, (2) the contig was between 10 and 45 kb in length, and (3) no virophage MCP was detected. The most abundant, complete (circular) putative PLV genome was then selected to be annotated by HHpred searches against the Protein Data Bank, where we identified an ATPase, minor capsid protein (mCP), and an MCP gene with distant homology to that of the giant virus *Paramecium bursaria Chlorella* virus 1 (PBCV-1), as has been reported for Polintons and Polinton-like viruses [3]. This confirmed MCP gene was then used to re-interrogate our dataset using BLASTP to identify further similar capsid genes (*E* value cutoff 10^−5^). We then repeated this procedure on the other putative PLV contigs (Additional file 1: Table S2), identifying a double jelly-roll fold capsid related to PBCV-1 or Faustovirus, and retrieving related genomes until we had either found a capsid or annotated via HHpred all virophage-like virus contigs. Finally, to further improve our detection of PLV in our metagenomes, we also gathered all contigs that contained an MCP gene homologous to any known Polinton or PLV from the literature (BLASTP *E* value < 10^−5^). In some cases, the MCP gene could still not be detected in a putative PLV after multiple searches; however, the presence of an ATPase and an mCP allowed us to carry these putative PLV contigs forward in the analysis and retrieve related contigs from our metagenomes using the mCP gene as above. Once we had clustered the viruses (see below), the MCP genes were then identified via a more sensitive HHpred query using alignments of core protein clusters. Contigs were confirmed as PLV when they met the following criteria: (1) 20% or more genes hit to a known or annotated virophage/PLV from Additional file 1: Table S1 or Additional file 1: Table S2 (BLASTP *E* value < 10^−5^), (2) contigs were between 10 and 41 kb, and (3) an MCP gene was found by HHpred. These settings were the result of extensive testing and annotation checks by HHpred to ensure no false positives were produced. As we retrieved viruses from multiple metagenomes, redundancy was removed from the dataset using CD-HIT [38] (Settings: psi-cd-hit.py -c 0.7 -G 1 -circle 1 -prog megablast) to produce a non-redundant PLV fasta file.

### Public metagenome searches

To determine if the Gossenköllesee PLVs were unique to this high mountain lakes, or part of a globally dispersed larger group of viruses, we interrogated large metagenomic datasets using the novel MCP genes as bait. The complete IMG/VR protein dataset [16] was downloaded (IMG_VR_2018-07-01_4; December 2018) along with all metadata (https://img.jgi.doe.gov/vr/). All known PLV MCP genes from GenBank and the literature (*n* = 25; Table 1) were pooled with all identified PLV MCPs from Gossenköllesee (*n* = 82) and aligned using MUSCLE [39]. A profile Hidden Markov Model (profile HMM) was built from the alignment using the hmmbuild command of HMMER (hmmer.org) and used to interrogate the downloaded IMG/VR protein dataset for PLV MCPs (hmmsearch --noali -E 0.00001). All matching records were retrieved from the IMG/VR fasta file (IMGVR_all_nucleatides.fna) as full contigs. Many IMG/VR PLV scaffolds, particularly circular ones, were redundant. These were removed via two rounds of clustering with CD-HIT (initial round settings: psi-cd-hit.py -c 0.9 -G 0 -aL 0.2 -circle 1 -prog megablast; second round: psi-cd-hit.py -c 0.7 -G 1 -circle 1 -prog megablast). Many IMG/VR PLV hits were detected in two large-scale sequencing projects from freshwater viromes from South Korea, Han River (PRJEB19373) and Lake Soyang (PRJEB15535) [17, 18]. To enhance the retrieval of viruses from these viromes, we downloaded all trimmed metagenomic reads from these two BioProjects from the European Bioinformatics Institute (EMBL-EBI; https://www.ebi.ac.uk/), re-assembled each individual metagenome, and cross assembled 3 metagenomes (PRJEB19373) using SPAdes (settings: --meta -k 21,33,53,77). PLVs were retrieved, made non-redundant, and confirmed as before.

To evaluate the relationships between PLVs and virophages, we also build a dataset of virophage genomes by concatenating known virophage genomes at the time of analysis (February 2019) with those retrieved from IMG/VR and other metagenomes (> 10 kb). To retrieve virophages from datasets, we used an identical method described for the PLV, building a separate profile HMM from the virophage MCP genes found in Gossenköllesee and 39 virophages from Table 1. This was before the release of a large virophage dataset mined from IMG/VR [13], but used similar methodology and searched the same database; hence, the virophages we retrieved from IMG/VR refer to those described in Paez-Espino et al. [13] (see Additional file 1: Table S4 for accession numbers). In total, 135 further virophages detected in this study were novel (Table 1).

### Network analysis

To assess the relatedness of the PLVs and virophages, we used a network-based approach to cluster virus sequences based on shared protein clusters (PCs) on each contig. Using all 1040 PLVs and virophages, protein clustering and generation of virus clusters (VCs) were performed by vConTACT v.2.03 [19] (settings: BLASTP 1e-4) on the CyVerse platform (http://www.cyverse.org/). The network was visualised using Cytoscape version 3.7.0 using an edge weighted forced spring embedded layout. The BLASTP protein clustering threshold was chosen to allow grouping of these distantly related viruses; however, an additional network analysis was conducted using stricter BLASTP protein-protein clustering thresholds, *E* value 10^−9^ (Additional file 2: Fig. S1) to assess the validity of the results.

### Protein cluster annotation

Owing to the novelty of the virus genomes in our dataset, most virus genes displayed little or no homology to known genes using BLASTP searches against the GenBank non-redundant protein database. More sensitive searches were carried out using Hidden Markov Model (HMM) comparisons to annotate protein clusters. The output from *vConTACT v.2.0* grouped the amino acid sequences from 1040 viruses into 2213 protein clusters based on sequence homology. These were annotated as follows: (1) members from each protein cluster were aligned using MUSCLE, (2) a profile HMM was built from each alignment using HMMER (hmmer.org), and (3) the profile was used to interrogate UNICLUST30 (uniclust30_2018_08) and Swiss-Prot (https://www.uniprot.org/) databases (HMMSEARCH *E* value cutoff 10^−4^). The top protein hit from each search was picked (excluding any hits to ‘Uncharacterized protein’) to create an annotation file (Additional file 1: Table S6). For the most abundant protein clusters and the core genes from each virus cluster, MUSCLE alignments were used to search the Protein Data Bank [14] (PDB_mmCIF70_10_Apr) using the HHpred server for remote protein homology [36].

### Phylogenetic analysis and eukaryotic host searches

MCP genes from Gossenköllesee were combined with those from known PLVs (*n* = 25) (Table 1), selected Polinton/PLV from oomycetes (*n* = 6), the *S. punctatus* element and Polintons from previous studies (*n* = 67) [3]. We also retrieved distantly related MCP homologues from GenBank nr using a PSI-BLAST search (*E* value < 10^−5^). When a GenBank hit was found, we confirmed this as an MCP by HHpred annotation before iterating the PSI-BLAST search. In this way, we detected MCP homologues (*n* = 27) in distantly related eukaryotic genomes. Alignment of PLV MCPs was by MAFFT version 7 (E-INS-i iterative refinement method) [40]. Maximum likelihood analysis was performed using PhyML [41] (LG substitution model with Shimodaira–Hasegawa-like estimation of branch support), and the tree was visualised in iTOL v4 (https://itol.embl.de/) using PBCV-1 as an outgroup.

We also compared all MCP genes detected in this study with eukaryotic transcriptomes from the Marine Microbial Eukaryotic Transcriptome Sequencing Project (MMETSP) [24]. The concatenated transcriptomes from this dataset were used as a DIAMOND BLASTX search [42] query against all PLV MCP genes (*E* value < 10^−10^), and genes were predicted on any contigs that hit to over 200 amino acids of an MCP gene (GeneMark.hmm; http://exon.gatech.edu/GeneMark/heuristic_gmhmmp.cgi) and annotated as an MCP by HHpred. Confirmed MCP genes from the transcriptomes were analysed by maximum likelihood phylogenetic analysis as above to place them into our defined virus groups.

## Supplementary Information

**Additional file 1: Table S1.** Virophages from Gossenköllesee annotated by HHpred. **Table S2.** PLVs from Gossenköllesee annotated by HHpred. **Table S3.** Polinton-like viruses and virophage genomes and genome fragments >10 KB from Gossenköllesee. **Table S4.** All Polinton-like virus and virophage metadata. **Table S5.** Virome hits to Silva database to assess contamination. **Table S6.** Protein clusters in each viral cluster. **Table S7.** Protein cluster annotations.

**Additional file 2: Fig. S1**. Higher stringency vConTACT2 network generated using BLASTP (E-value cutoff 10^-9^) protein-protein comparisons**.** See Fig. 3 for full description.

## Data Availability

The datasets supporting the conclusions of this article are available in the figshare repository https://figshare.com/s/9a7a1d16d77ea9d658a1. This includes all nucleotide sequences from the PLV and virophages sequenced in this study from Gossenkӧllesee plus those reassembled from EBI metagenomes, all amino acid gene predictions used for virus clustering, vConTACT protein clusters, alignments of MCP genes, and profile HMMs for PLV and virophage MCPs. All IMG/VR-derived sequences are listed with scaffold identification numbers in Additional file 1: Table S4, and these are available for download at https://img.jgi.doe.gov/vr/. The European Bioinformatics Institute metagenomes are available at https://www.ebi.ac.uk/metagenomics/ using the BioProject IDs in Table 1.

## Abbreviations

CroV: *Cafeteria roenbergensis virus*
IMG/VR: Integrated Microbial Genomes Virus
MCP: *Major capsid protein*
mCP: *Minor capsid protein*
MMETSP: Marine Microbial Eukaryotic Transcriptome Sequencing Project
NCLDV: Nucleocytoplasmic large DNA virus
PBCV: *Paramecium bursaria Chlorella virus*
PC: Protein cluster
PgVV: *Phaeocystis globosa virus virophage*
PLV: *Polinton-like virus*
pPolB: Protein-primed type B DNA polymerase
RVE: Retroviral integrase
TIR: Terminal inverted repeat
