# Correction to: Polinton-like viruses are abundant in aquatic ecosystems

**DOI:** 10.1186/s40168-021-01004-1

**Published:** 2021-01-27

**Authors:** Christopher M. Bellas, Ruben Sommaruga

**Affiliations:** Department of Ecology, University of Innsbruck, Technikerstrasse 25, A-6020 Innsbruck, Austria

**Correction to: Microbiome 9, 13 (2021)**

**https://doi.org/10.1186/s40168-020-00956-0**

Following publication of the original article [1], it has been reported that reference 5 author group was incorrectly captured.

Reference 5 is currently captured as:

Fischer MG, C a S. A virophage at the origin of large DNA transposons. Science. 2011;332:231–4.

Reference 5 should be captured as:

Fischer MG, Suttle CA. A virophage at the origin of large DNA transposons. Science. 2011;332:231–4.

The original article [1] has been corrected.
