# Supplementary figures and images for: Polinton-like viruses are abundant in aquatic ecosystems
Source: Microbiome. 2021 Jan 12;9:13. doi: 10.1186/s40168-020-00956-0 (PMC7805220; doi:10.1186/s40168-020-00956-0)

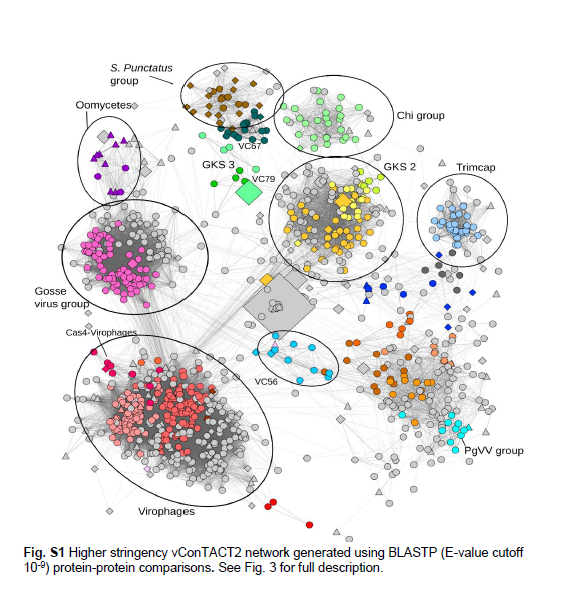

Supplement: Supplementary file 3 — Additional file 2: Fig. S1. Higher stringency vConTACT2 network generated using BLASTP (E-value cutoff 10-9) protein-protein comparisons. See Fig. 3 for full description. [file 40168_2020_956_MOESM3_ESM.png]
